# Effects of recombinant human growth hormone (rhGH) administration on body composition and cardiovascular risk factors in obese adolescent girls

**DOI:** 10.1186/1687-9856-2014-22

**Published:** 2014-11-15

**Authors:** Meghan Slattery, Miriam A Bredella, Takara Stanley, Martin Torriani, Madhusmita Misra

**Affiliations:** Neuroendocrine Unit, Massachusetts General Hospital and Harvard Medical School, BUL 457B, Neuroendocrine Unit, 55 Fruit Street, MGH, Boston, MA 02114 USA; Department of Radiology, Massachusetts General Hospital and Harvard Medical School, Boston, MA 02114 USA; Pediatric Endocrine Unit, Massachusetts General Hospital for Children and Harvard Medical School, Boston, MA 02114 USA

**Keywords:** Adolescents, Growth hormone, Visceral fat, Body composition, Females, Inflammatory markers

## Abstract

**Background:**

Obesity is associated with a relative deficiency of growth hormone, which is predictive of greater visceral fat and markers of cardiovascular risk. The study’s purpose was to use recombinant human growth hormone (rhGH) as a physiologic probe to assess the effects of reversing obesity-related GH deficiency on body composition, cardiovascular risk markers, and insulin resistance.

**Methods:**

22 obese girls 13–21 years old were followed for a randomized 6-month trial of rhGH vs. placebo/no treatment. At baseline and 6-months, DXA was performed for body composition, MRI to measure visceral, subcutaneous and total adipose tissue (VAT, SAT and TAT), and fasting blood drawn for IGF-1, inflammatory cardiovascular risk markers [soluble intercellular adhesion molecule (sICAM), high sensitivity CRP], lipids and HbA1C. An oral glucose tolerance test (OGTT) was performed. Twelve girls completed the 6-month visit. Baseline and mean 6-month change were compared between the groups using the Student *t*-test and the relationship between variables was determined through multiple regression analysis.

**Results:**

After 6-months, the rhGH group maintained IGF-1 levels, and had decreases in total cholesterol (p = 0.03), sICAM-1 (p = 0.04) and HbA1C (p = 0.03) compared to placebo/no treatment. The rhGH group trended towards greater decreases in LDL and 2-hour OGTT glucose. Glucose tolerance did not worsen with rhGH administration.

**Conclusions:**

Administering rhGH in small doses is able to stabilize IGF-1 levels in obesity. We have also shown that rhGH administration leads to an improvement in some markers of cardiovacular risk with without adversely affecting glucose tolerance.

**Trial registration:**

Clinical Trial Registration Number: NCT01169103.

## Background

Obesity, a pressing global issue, is characterized by diminished growth hormone (GH) secretion in adults [1, 2] and adolescents [3], with decreased frequency and amplitude of GH secretory bursts [4]. Pathological GH deficiency is characterized by a high risk cardiovascular profile [5–8], and similarly, in obese individuals, relatively low GH levels are associated with higher visceral fat [3], which in turn predisposes individuals to components of the metabolic syndrome [9, 10], including hyperlipidemia [10, 11] and insulin resistance [10, 12]. While GH replacement in children with pathologic GH deficiency causes a decrease in visceral adiposity [13], effects of GH administration on body composition have not been examined in an otherwise healthy adolescent obese population that is relatively GH insufficient.

Existing studies examining the effects of rh(GH) on obesity and cardiovascular risk factors have largely been performed either on adults or in children with specific chronic health conditions such as GH deficiency [13] or Prader-Willi Syndrome [14]. Consequently, there is a dearth of data regarding effects of GH replacement on body composition and cardiometabolic risk in otherwise healthy obese adolescents. This proof-of-concept study used rhGH as a physiologic probe to observe the effects of GH replacement on body composition. We hypothesized that rhGH administration in replacement doses to obese adolescent females would have lipolytic effects without deleterious effects on glucose tolerance.

## Methods

The study was approved by the Institutional Review Board of Partners HealthCare system. Written informed consent (for patients ≥18 years) or parental consent with participant assent (for patients <18 years) was obtained from all.

### Subject selection

Participants were recruited at Massachusetts General Hospital (MGH) between September 2010 and October 2012 through area pediatric and obesity clinics. Of 32 girls and young women 13–21 years old assessed for study eligibility, 22 obese adolescents met inclusion criteria and were randomized. Inclusion criteria comprised (i) a bone age ≥14 years, (ii) body mass index (BMI) > 95th percentile for age (based on the 2000 Centers for Disease Control and Prevention Growth Charts) [15], or >30 kg/m^2^ if age >18 years, (iii) insulin like growth factor-1 (IGF-1) level below the median for pubertal stage or age, (iv) abdominal obesity with a waist to hip ratio (W/H) >0.85, and (v) stable weight (<5 kg change in weight in the prior 3 months). Exclusion criteria included diabetes mellitus, untreated thyroid dysfunction, chronic renal insufficiency, current or past malignancy, syndromic obesity, pregnancy, breast-feeding, and use of medications known to alter glucose metabolism or body composition (contraceptive pills, daily glucocorticoid use, metformin, sibutramine and Orlistat). Additionally, we excluded girls with new (<6 months) or unstable dosing (dosage change within 3-months) of antipsychotic medications known to cause weight gain. Baseline characteristics for a subset of study participants have been previously published [16].

Eleven subjects were randomized to active rhGH treatment {rhGH (+)} and 11 to rhGH negative {rhGH (-)} treatment. Sixteen subjects {10 rhGH (+) and 6 rhGH (-)} completed the 3-month visit, and 12 subjects {5 rhGH (+) and 7 rhGH (-)} finished the 6-month study period (one rhGH (-) subject missed the 3-month visit but completed other visits). No subject dropped out because of side effects. Of subjects who withdrew (n = 10), one chose to begin an oral contraceptive, 3 withdrew because of personal obligations, and the remaining were considered lost to follow-up (n = 6). Study participants who did or did not complete the study did not differ for baseline characteristics.

### Experimental protocol

The study was a 6-month, single-blind, randomized trial, conducted at the Clinical Research Center of MGH. The screening visit included a history and physical examination and blood sampling to ensure participants met inclusion criteria and to rule out exclusion criteria. Diabetes was ruled out via an oral glucose tolerance test (OGTT). Eligible subjects returned for the baseline visit, visits at 1 and 2 weeks (for dose adjustment), and at 1, 3 and 6 months. Subjects were randomized 1:1 to receive once daily subcutaneous rhGH or placebo injections (Somatropin, Genentech, Inc., San Francisco, CA, USA), and taught to self inject medication daily for 6-months. Due to non-availability of placebo (and inability for new placebo to be manufactured) after June 2012, subjects subsequently randomized to placebo (n = 4) were instead randomized to no treatment. Dieticians and the radiologist who performed study related procedures as well as all subjects who enrolled prior to June 2012 (N = 15) remained blinded to randomization status for the entirety of the study. After the placebo expiration necessitated the change in the study design, one subject randomized to rhGH (+) subject and one subject randomized to rhGH (-) treatment dropped out. The subject randomized to rhGH (-) treatment withdrew due to the initiation of an oral contraceptive while the subject randomized to rhGH (+) was lost to follow up after the 3 month visit.

The starting rhGH dose was 0.4 mg and increased at week-1 and week-2 to 0.6 mg and 0.8 mg respectively. The dosing was based on a dose of 12.5 mcg/kg/day for a 16-year-old girl (mean for bone ages 14–18 years) weighing 67 kg (85th percentile for age) [17]. This dosage (0.8 mg at week-2) is at the lower end of the 12.5–25 mcg/kg/day dose used in studies of GH deficient adolescents transitioning from pediatric (40–45 mcg/kg/day) to adult (2–6 mcg/kg/day) replacement rhGH doses [17, 18]. We opted for this lower dose as we expected lower GH requirements in this population that is relatively, rather than completely, GH insufficient. The dose was adjusted as needed by 20% at the 1 and 3-month visits to achieve IGF-1 levels in the upper half of the normal range for pubertal stage. When target IGF-1 was achieved, the individualized dose was continued.

### Study procedures

Height was measured as the average of three measurements to the nearest 0.1 cm on a single calibrated wall-mounted stadiometer. Participants, wearing a hospital gown, were weighed to the nearest 0.1 kg on a single calibrated electronic scale. BMI was calculated as weight (in kg) divided by height (in meters^2^) and BMI standard deviation scores (SDS) determined from 2000 CDC charts [15]. Waist measurements were taken with a plastic tape measure to the nearest 0.1 cm at the level of the iliac crest and umbilicus; the maximum hip circumference was measured. All measurements were taken at the end of expiration with the subject standing. Waist-to-hip ratio (W/H) was calculated as the iliac divided by the hip circumference measurement. An ophthalmoscope was used to rule out papilledema at study onset, and at the 3 and 6-month visits, or if any subject complained of headaches, vision changes, nausea or vomiting. Subjects had a left hand x-ray to assess bone age [19]. Pubertal stage was determined according to the criteria of Tanner [20].

Magnetic resonance imaging (MRI) at the level of L4 was used to determine visceral adipose tissue (VAT) and subcutaneous adipose tissue (SAT) at baseline and 6 months; total abdominal adipose tissue (TAT) was calculated as VAT + SAT [21]. MRI data are available for 11 completers (due to scheduling conflicts one no-treatment subject was unable to perform the MRI component). Body composition was also obtained by dual energy x-ray absorptiometry scans (DXA) at the baseline, 3 and 6-month visits. DXA (Hologic QDR-Discovery A; Hologic Inc., Waltham, MA software version APEX 3.3) was used to assess percent body fat, total, trunk and extremity fat and total and extremity lean mass.

Subjects met with nutritionists of the Clinical Research Center at the baseline, 3 and 6-month visits for (i) basic lifestyle counseling including healthy eating habits and optimizing exercise, and (ii) assessment of prior activity using the Modified Activity Questionnaire (MAQ) [22]. To avoid confounding variables, further dietary or exercise restrictions were not imposed.

Fasting blood samples were drawn for IGF-1 at each visit for rhGH dose adjustment, and a 75-g, 2-hour OGTT performed at screen, 3 and 6-months to determine whether rhGH administration had deleterious effects on glucose tolerance. Fasting blood samples were also assessed for cardiovascular risk markers [lipids, high sensitivity C-reactive protein (hs-CRP), soluble intercellular adhesion molecule-1 (sICAM-1)], HbA1C, glucose and insulin levels at baseline and 6-months.

IGF-1 was analyzed by enzyme-linked immunosorbent assay (ELISA) (Immunodiagnostic systems, Scottsdale, AZ; Limit of Detection (LOD) 3.1 ng/mL, CV < 7%). Fasting insulin was analyzed by immunoassay (Cobas, Roche Diagnostics, Indianapolis, IN; LOD 0.2 μU/mL, CV 0.8 to 4.9%), glucose via an enzymatic *in vitro* test (Cobas, Roche Diagnostics, Indianapolis, IN; LOD 2 mg/dL, intra-assay CV 1.0%), total cholesterol, LDL and HDL via a Roche direct assay (Cobas, Roche Diagnostics, Indianapolis, IN) [total cholesterol (LOD 3 mg/dL, CV 0.8–1.0%), LDL (LOD 3 mg/dL, intra-assay CV 0.71–1.22%) and HDL (LOD 3 mg/dL, intra-assay CV 0.60–0.95%)], triglycerides via the Roche triglyceride assay (Cobas, Roche Diagnostics, Indianapolis, IN; LOD 4 mg/dL, intra-assay CV 0.9–1.5%), and VLDL calculated by subtracting LDL and HDL from total cholesterol. sICAM-1 was analyzed by ELISA (RnD Systems, Minneapolis, MN; minimum detectable concentration 1 ng/mL, CV < 8%), hsCRP via an immunoradiometric assay (IRMA) (LabCorp, Burlington, NC; LOD 0.3 mg/L, intra-assay CV < 10%). ALT was analyzed by the Architect assay (Abbott, Abbott Park, IL; LOD 2.0 U/L, intra-assay CV < 5.2%). Serum was stored at -80°C for insulin, ALT, and cardiovascular risk factors until analysis. Other samples were analyzed in real time.

### Statistical methods

JMP Software (v10: SAS Institute, Inc., Cary, NC) was used for analysis. Results are reported as means ± SD. Baseline and mean 6-month change were compared using the Student *t*-test or Wilcoxon rank sum test depending on data distribution. A paired *t*-test was also used to compare baseline and 6 month values within groups. Parametric (Pearson) or nonparametric (Spearman) correlations were used as appropriate to determine associations between variables that were or were not normally distributed. Significance was defined as a two-tailed p-value of <0.05. Multivariate regression models were constructed to determine whether significances for changes in endpoints persisted after controlling for weight changes or baseline values of the endpoint (i.e. HbA1c).

## Results

### Baseline characteristics

Baseline characteristics are summarized in Table 1. Study participants had a mean age of 16.6 ± 2.4 years, mean weight of 100.1 ± 20.5 kg, and a mean BMI of 38.5 ± 7.5 kg/m^2^. Treatment groups did not differ for baseline characteristics except for a slightly lower HbA1C in the rhGH (-) group. There were no significant baseline differences noted between the blinded (N = 15) and unblinded (N = 7) groups with one exception; the W/H ratio was higher in the group who remained blinded to their treatment allocation (0.96 ± 0.06 vs. 0.90 ± 0.07, p = 0.05). All subjects were Tanner Stage V for breast development at the baseline visit.

**Table 1:** **Baseline characteristics of all obese adolescent girls at study entry**

	RhGH + N = 11	RhGH - N = 11	P
Age (years)	16.2 ± 2.6	16.9 ± 2.2	0.50
Bone age (years)	16.9 ± 1.2	17.2 ± 1.3	0.56
Weight (kg)	104.7 ± 24.8	97.4 ± 15.4	0.42
BMI (kg/m^2^)	40.4 ± 8.4	36.6 ± 6.4	0.25*
BMI SDS	2.3 ± 0.4	2.1 ± 0.4	0.22
Waist circumference (cm)	119.4 ± 17.1	115.0 ± 14.8	0.53
W/H ratio	0.95 ± 0.06	0.93 ± 0.08	0.62
SAT (cm^2^)	615.6 ± 175.0	580.0 ± 176.9 (N = 9)	0.66
VAT (cm^2^)	90.6 ± 35.3	87.1 ± 31.8 (N = 9)	0.82
TAT (cm^2^)	706.2 ± 203.5	667.2 ± 179.8 (N = 9)	0.66
Thigh subcutaneous fat (cm^2^)	224.2 ± 69.4	221.4 ± 96.3	0.95
Lean mass (grams)	54353 ± 9719	52276 ± 6842	0.57
IGF-1 (ng/mL)	250.6 ± 129.9	271.8 ± 73.1	0.64
2 HR glucose (OGTT) (mg/dL)	107.1 ± 20.5	114.0 ± 18.5	0.42
HbA1c (%)	5.78 ± 0.30	5.49 ± 0.30	**0.03**
Insulin uU/mL	26.4 ± 21.3	40.1 ± 37.2	0.29*
Total cholesterol (mg/dL)	167.6 ± 38.3	156.0 ± 33.5	0.46
LDL (mg/dL)	103.8 ± 28.8	92.6 ± 30.1	0.33*
HDL (mg/dL)	45.1 ± 9.4	43.3 ± 8.5	0.64
VLDL (mg/dL)	18.6 ± 9.4	20.1 ± 8.0	0.70
Triglycerides (mg/dL)	93.0 ± 47.0	100.6 ± 39.8	0.69
hsCRP (mg/L)	4.47 ± 2.76	3.41 ± 3.29	0.25*
sICAM-1 (ng/mL)	268.7 ± 72.5	220.9 ± 56.4	0.10

*P value reported for log transformed values.

RhGH+: Group that received rhGH; RhGH-: Group that received placebo/no treatment.

*Abbreviations*: *BMI* body mass index, *BMI SDS* body mass index standard deviation score, *HbA1c* hemoglobin A1c, *HDL* high density lipoprotein, *hsCRP* high sensitivity C-reactive protein, *IGF-1* insulin like growth factor-1, *LDL* low density lipoprotein, *OGTT* oral glucose tolerance test, *SAT* subcutaneous adipose tissue, *sICAM-1* soluble intercellular adhesion molecule 1, *TAT, VAT* total, visceral adipose tissue, *VLDL* very low density lipoprotein, *W/H* waist to hip ratio.

### Six-month changes in nutritional measures

No differences were noted between rhGH(+) and rhGH(-) groups for changes in calories consumed (142.6 ± 961.0 vs. 193.5 ± 644.0 kcal, p = 0.93), percentage of calories from fat (-8.09 ± 12.04 vs. 0.09 ± 12.95%, p = 0.36), or activity levels (1.64 ± 6.4 vs. 8.83 ± 9.48 hours/week, p = 0.18).

### Six-month changes in IGF-1 levels

Changes in IGF-1 trended higher in the treatment group (Table 2). At the 6-month visit, mean IGF-1 in the rhGH(+) group was non-significantly higher than in the rhGH(-) group (234 ± 24.3 vs. 185.6 ± 20.5 ng/mL, p = 0.16).

**Table 2:** **Baseline, 6-month and change in body composition and biochemical parameters over 6-months for study completers**

		RhGH +		RhGH -			
	Baseline	6-month N = 5	6-month delta	Baseline	6-month N = 7	6-month delta	6 month delta P
Weight (kg)	99.9 ± 29.5	100.6 ± 30.9	0.7 ± 4.3	96.5 ± 18.1	100.4 ± 23.1	3.9 ± 6.6	0.81*
BMI (kg/m^2^)	39.6 ± 10.2	39.8 ± 10.8	0.2 ± 2.0	36.4 ± 7.3	37.8 ± 9.6	1.4 ± 2.5	0.81*
Waist circumference (cm)	115.4 ± 21.7	113.7 ± 23.1	-1.7 ± 4.0	112.4 ± 17.0	112.5 ± 21.2	0.1 ± 6.5	0.60
W/H ratio	0.94 ± 0.06	0.95 ± 0.06	0.003 ± 0.03	0.91 ± 0.08	0.91 ± 0.08	-0.005 ± 0.04	0.72
SAT (cm^2^)	578.5 ± 234.8	577.4 ± 232.9	-1.1 ± 60.8	570.0 ± 206.4	606.3 ± 231.1	36.3 ± 51.6 (N = 6)	0.30
VAT (cm^2^)	92.7 ± 36.6	93.3 ± 47.3	0.6 ± 29.9	90.4 ± 39.6	98.4 ± 43.5	8.0 ± 31.9 (N = 6)	0.70
TAT (cm^2^)	671.3 ± 264.1	670.7 ± 278.9	-0.5 ± 83.3	660.3 ± 209.1	704.6 ± 234.5	44.3 ± 63.1 (N = 6)	0.34
Thigh subcutaneous fat (cm^2^)	217.7 ± 72.1	208.7 ± 73.1	-9.0 ± 16.0	228.5 ± 103.4	211.2 ± 72.7	18.6 ± 29.0 (N = 6)	0.08*
Lean mass (grams)	50741.3 ± 7963.2	50758.1 ± 8735.6	17 ± 2160	50659.5 ± 7149.8	51360.7 ± 7309.6	701 ± 2325	0.62
IGF-1 (ng/mL)	235.4 ± 61.9	234.0 ± 48.5	-1.4 ± 79.4	266.0 ± 83.6	185.9 ± 58.0	-80.1 ± 48.8	0.06
2 HR-glucose (OGTT) (mg/dL)	119.8 ± 14.8	113.2 ± 17.6	-6.6 ± 17.0	112.7 ± 14.2	135.1 ± 23.7	22.4 ± 28.8	0.07
HbA1c (%)	5.62 ± 0.30	5.58 ± 0.16	-0.04 ± 0.17	5.47 ± 0.25	5.61 ± 0.30	0.14 ± 0.08	**0.03**
Insulin uU/mL	25.4 ± 25.7	22.6 ± 17.8	-2.8 ± 11.7	41.9 ± 41.0	27.1 ± 22.5	-14.8 ± 38.3	0.94*
Total cholesterol (mg/dL)	189.2 ± 42.6	151.4 ± 27.8	-37.8 ± 23.9	162.1 ± 39.7	153.6 ± 34.8	-8.6 ± 15.5	**0.03**
LDL (mg/dL)	121.2 ± 27.7	95.4 ± 22.4	-25.8 ± 12.8	98.3 ± 35.8	87.6 ± 28.0	-10.7 ± 11.9	0.06
HDL (mg/dL)	48.2 ± 13.4	41.6 ± 8.2	-6.6 ± 6.1	44.0 ± 7.4	45.0 ± 5.2	1.0 ± 5.1	**0.04**
VLDL (mg/dL)	19.8 ± 11.2	14.4 ± 6.0	-5.4 ± 9.6	19.9 ± 7.0	21.0 ± 16.3	1.1 ± 11.7	0.48*
Triglycerides (mg/dL)	99.4 ± 55.7	71.6 ± 29.5	-27.8 ± 46.8	99.1 ± 35.5	105.3 ± 24.9	6.1 ± 58.4	0.57*
hsCRP (mg/L)	3.5 ± 2.1	2.69 ± 1.8	-0.77 ± 2.36	4.4 ± 3.8	4.27 ± 4.6	-0.09 ± 1.79	0.58
sICAM-1 (ng/mL)	226.4 ± 79.3	204.2 ± 74.5	-22.2 ± 30.3	241.1 ± 48.1	262.2 ± 74.8	21.1 ± 33.3	**0.04**

*Wilcoxon rank sum test.

HbA1c, Total Cholesterol, HDL and sICAM were lower after 6 months of rhGH + treatment.

RhGH+: Group that received rhGH; RhGH-: Group that received placebo/no treatment.

Baseline: Mean Baseline values only for study completers.

6-month: Mean 6-month values for study completers.

6-month delta: Change over 6-months from Mean Baseline to Mean 6-month values.

*Abbreviations*: *BMI* body mass index, *BMI SDS* body mass index standard deviation score, *HbA1c* hemoglobin A1c, *HDL* high density lipoprotein, *hsCRP* high sensitivity C-reactive protein, *IGF-1* insulin like growth factor-1, *LDL* low density lipoprotein, *OGTT* oral glucose tolerance test, *SAT* subcutaneous adipose tissue, *sICAM-1* soluble intercellular adhesion molecule 1, *TAT, VAT* total, visceral adipose tissue, *VLDL* very low density lipoprotein, *W/H* waist to hip ratio.

### Six-month changes in body composition

**Figure 1:**
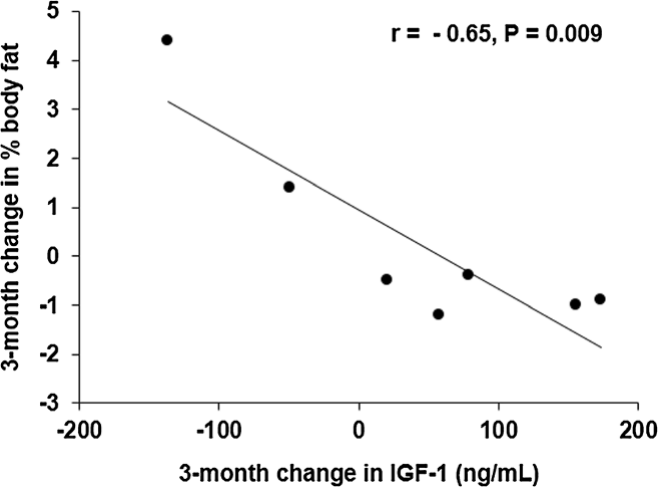
**Correlation between 3-month changes in IGF-1 and body fat percentage within the rhGH treated group.** 3-month increase in IGF-1 was associated with the 3-month decrease in % body fat.

Body composition changes across treatment groups are presented in Table 2. Although changes in waist circumference and W/H ratio did not differ across groups over the study duration, the increase in IGF-1 between 3–6 months correlated with the decrease in W/H ratio over 6-months (r = -0.74 p = 0.009). A similar finding was also seen in the negative correlation between change in IGF-1 over 3-months and change in body fat percentage over 3-months (r = -0.65 p = 0.009) (Figure 1). SAT and TAT decreased non-significantly in the treatment group compared to an increase in the control group, and VAT showed a negligible increase compared to the control group.

### Six-month changes in cardiovascular risk markers

#### Lipid levels

**Figure 2:**
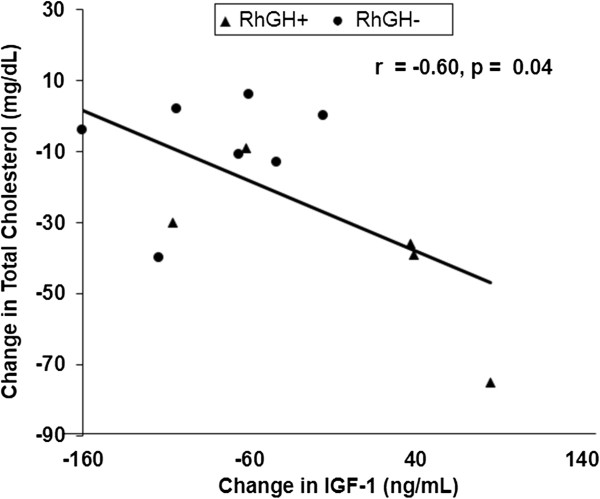
**Correlation of 6-month change in IGF-1 with 6-month change in total cholesterol for all subjects.** 6-month increase in IGF-1 was associated with the 6-month decrease in total cholesterol.

At 6-months, subjects in the active group had significant reductions in total cholesterol compared with controls (Table 2), and trended to have greater reductions in LDL. The treatment group also had a greater decrease in HDL compared with controls. Six-month changes in IGF-1 correlated negatively with changes in total cholesterol (r = -0.60, p = 0.04) (Figure 2), VLDL (Spearman rho = -0.58, p = 0.05), and triglycerides (Spearman rho = -0.67, p = 0.02) but not HDL (r = -0.37, p = 0.24).

#### Markers of inflammation

**Figure 3:**
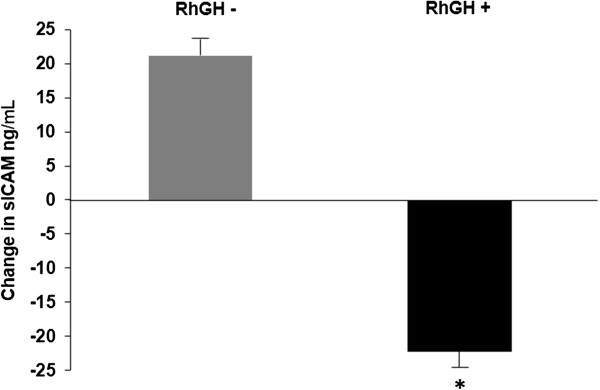
**Change in soluble intercellular adhesion molecule-1 (sICAM) levels in obese adolescent females.** Change in sICAM levels in obese adolescent females after 6 months of recombinant human growth hormone therapy (rhGH +) (black bar) or placebo/no treatment (rhGH -) (gray bar).

Subjects in the treatment group had a significant decrease in sICAM-1 (a marker of inflammation) compared to an increase in controls (Table 2 and Figure 3). Mean changes in hsCRP did not differ across groups. Additionally, there was a trend for GH to decrease ALT, a marker of liver inflammation, (-5 ± 5 vs. 3 ± 11 U/L, p = 0.18 for a 2-tailed test and 0.0478 for a one-tailed test).

### Six-month changes in glycemic status

At 6-months, subjects in the active group had significant reductions in HbA1c compared to controls (Table 2). This reduction trended to remain significant after controlling for baseline HbA1C (p = 0.06). Subjects in the active group also trended to have greater reductions in 2-hour glucose levels on the OGTT (Table 2). No difference was noted between the groups for 6-month changes in insulin levels. Thus, we did not see the anticipated deleterious effects of rhGH on glucose tolerance.

### Adverse events over the 6-month study duration

Adverse events are presented in Table 3. There were no serious adverse events related to study participation. rhGH was well tolerated and no subject required a dose reduction. No subject who complained of headaches, nausea or vomiting had a history or physical exam findings suggestive of pseudotumor cerebri.

**Table 3:** **Adverse events in treated obese girls (rhGH+) and untreated obese girls (rhGH -)**

Event type	Total number of adverse events	Adverse events	
		RhGH + (N)	RhGH - (N)
**Serious adverse events**	-	-	-
**Non-serious adverse events**			
*Hyperglycemia related events*			
IGT at 3 or 6-month (OGTT)	6	2	4
Polyuria/Polydipsia	4	3	1
HbA1c > 6.4%	1	1	-
*Symptoms that may relate to raised ICP*			
Headache	7	4	3
Nausea with vomiting	1	1	-
Nausea without vomiting	3	3	-
Dizziness without headache	1	-	1
Blurry vision	1	1	-
*Arthralgias/Fluid retention related events*			
Arthralgia	2	2	-
Back pain	5	4	1
Myalgia	1	1	-
Edema	-	-	-
*Menstrual cycle related events*			
Change in menstrual Flow	5	3	2
*Injection related events*			
Bruising/Irritation at injection site	4	2	2
Bruising/Irritation at blood sampling site	1	1	-
*Others*			
Hypertension	1	-	1
Lightheadedness	-	-	-
ED visit for wheezing with URI	1	-	1
Abdominal pain	2	2	-
Upper respiratory Infection	1	1	-
Nasal congestion (with URI)	1	1	-
Fatigue	2	-	2
Eczema	1	-	1

RhGH+: Group that received rhGH; RhGH-: Group that received placebo/no treatment.

*Abbreviations*: *ED* emergency department, *ICP* intracranial pressure, *IGT* impaired glucose tolerance, *OGTT* oral glucose tolerance test, *URI* upper respiratory infection.

There were no significant differences across the two groups for the various adverse events.

## Discussion

We have shown that administering rhGH to female adolescents in physiologic doses is able to stabilize IGF-1 in obesity, a state characterized by relative GH insufficiency, without adversely affecting glucose tolerance. We also confirmed the lipolytic effects of endogenous rhGH, administered for the first time to otherwise healthy obese adolescent females, resulting in a decrease in total cholesterol; additionally, we saw a reduction in sICAM-1 as opposed to an increase in sICAM-1 levels in the non-intervention group. Our group has previously reported a relative state of GH insufficiency in obese adolescent girls compared with controls, and that lower GH levels strongly predict higher visceral fat, an important determinant of insulin resistance and hyperlipidemia [3]. Although GH replacement causes a decrease in visceral adiposity in GH deficient children [13], effects of GH administration on body composition and cardiometabolic risk have not been previously examined in a healthy adolescent obese population.

Despite small increases in IGF-1, the dose of rhGH used in our study achieved significant reductions in total cholesterol and sICAM-1. It is thus possible that observed changes represent direct lipolytic effects of GH and direct effects of GH on inflammatory markers, rather than IGF-1 mediated effects. However, we did observe significant inverse associations between 6-month changes in IGF-1 levels and 6-month changes in total cholesterol, triglycerides, and VLDL. Although we observed a decrease in HDL levels in the active arm, no correlation was found between changes in IGF-1 levels and changes in HDL over the 6-month study period.

Of note, by the end of puberty, the increase in insulin resistance that is characteristic of puberty resolves, and returns to prepubertal levels [23]. All subjects in our study were fully pubertal at the start of the study, thus any puberty related variations in insulin resistance parameters were minimized. We speculate that the decrease in sICAM levels in the active arm compared with the increase in the non-treatment arm may represent reduced inflammation in the active arm, compared to a persistent (and potentially worsening) pro-inflammatory state in the non-treatment arm. This may account for the maintenance of HbA1C levels in the intervention group, as opposed to the increase in HbA1C observed over 6 months in the non-intervention group. Given that rhGH studies in adults have found that even at low doses, GH treatment corresponds with elevated glucose and insulin levels [5], adolescence may potentially offer a time period when rhGH replacement (in doses used in this study) will not worsen glucose tolerance.

Given the known inverse associations of GH levels with VAT and hsCRP in obesity, [3, 24, 25] we expected to see a reduction in VAT content and in hsCRP levels following rhGH administration. However, contrary to our expectations, neither of these parameters decreased, and it is possible that higher doses of rhGH than achieved in this study are necessary to observe such an effect. Following rhGH administration, although the mean IGF-1 level attained in the active arm was higher than in the control arm, this was not statistically significant, and mean levels remained in the lower half of the normal range despite dose titration. Thus, a state of relative GH insufficiency persisted. It is unclear why IGF-1 decreased in the control group. Although variation within the IGF-1 range is an expected finding, it is also possible that the non-significant weight gain in the control group over 6 months contributed to a decrease in GH, and therefore, IGF-1 secretion [3, 24, 25].

We did observe inverse associations of changes in IGF-1 with changes in the waist/hip ratio, considered to be an excellent surrogate for VAT, which also suggests that a higher dosage of rhGH may have caused improvements in body composition and biochemical parameters overall. The fact that glucose tolerance did not deteriorate in the active arm may also be attributable to the low dosage of rhGH used in the active arm [24, 26]. Further studies are necessary based on data from this proof-of-concept study to determine the most appropriate dose of rhGH to improve body composition and cardiometabolic risk markers in obese adolescent girls without significantly worsening insulin resistance.

Limitations of this study include the small sample size and relatively high drop-out rate (common in studies with obese subjects) [27–29]. However, a significant strength was that we observed changes in lipids and an inflammatory marker despite the small sample size and low GH dose. Many previous studies examining the relationship between IGF-1 and body composition limited their findings to BMI [30, 31] and previous prospective rhGH treatment studies focused on obese adults [2, 24, 25]. The remaining studies that either prospectively or retrospectively analyzed the effects of rhGH on body composition in the pediatric population involved children with Prader-Willi syndrome or GH deficiency [13, 14, 32]. This current study is the first to examine the effects of rhGH on otherwise healthy obese adolescents.

## Conclusions

In conclusion, we have demonstrated that low rhGH doses are easily tolerated with minimal side effects and are able to stabilize IGF-1 levels in obese adolescent girls. Our study confirms the lipolytic effects of growth hormone and suggests that rhGH replacement in viscerally obese adolescent females reduces total cholesterol and sICAM without adversely affecting glycemic status. Further studies are necessary to confirm these findings, and to also better understand the potential clinical role of rhGH as a treatment for obese adolescent girls.

## Abbreviations

BMI: Body mass index
CV: Coefficient of variation
DXA: Dual energy x-ray absorptiometry scan
ELISA: Enzyme-linked immunosorbent assay
FAI: Free androgen index
GH: Growth hormone
HbA1c: Hemoglobin A1c
HDL: High density lipoprotein
HOMA-IR: Homeostasis model assessment of insulin resistance
hs-CRP: High-sensitivity C-reactive protein
IGF-1: Insulin-like growth factor 1
IRMA: Immunoradiometric assay
LDL: Low density lipoprotein
LOD: Limit of detection
MAQ: Modified activity questionnaire
MGH: Massachusetts General Hospital
MRI: Magnetic resonance imaging
OGTT: Oral glucose tolerance test
(rh)GH: Recombinant human growth hormone
SAT: Subcutaneous abdominal adipose tissue
SD: Standard deviation
SDS: Standard deviation score
SHBG: Sex hormone binding globulin
sICAM-1: Soluble intercellular adhesion molecule-1
TAT/VAT: Total/visceral abdominal adipose tissue
VLDL: Very low density lipoprotein
W/H: Waist to hip ratio.
